# Rendu-Osler-Weber Syndrome: case report and literature review

**DOI:** 10.1016/S1808-8694(15)30582-6

**Published:** 2015-10-19

**Authors:** Antônio José Cortez Juares, Alfredo Rafael Dell’Aringa, José Carlos Nardi, Kazue Kobari, Vera Lúcia Muller Gradim Moron Rodrigues, Renato Martins Perches Filho

**Affiliations:** 1 Resident Physician.; 2 PhD in Otorhinolaryngology, Head of the Otorhinolaryngology Department - Faculdade de Medicina de Marília.; 3 MSc in Otorhinolaryngology. Associate Professor of Otorhinolaryngology Faculdade de Medicina de Marília.; 4 MD. Otorhinolaryngologist. Professor of Otorhinolaryngology Faculdade de Medicina de Marília.; 5 MD. Otorhinolaryngologist. Volunteer Professor of Otorhinolaryngology Faculdade de Medicina de Marília.; 6 MD. Otorhinolaryngologist. Former ENT Resident from the Faculdade de Medicina de Marília. Disciplina de Otorrinolaringologia. Faculdade de Medicina de Marília.

**Keywords:** epistaxis, rendu-osler-weber syndrome, hereditary hemorrhagic telangiectasia

## Abstract

Hereditary Hemorrhagic Telangiectasia or Rendu-Osler-Weber Disease is a rare fibrovascular dysplasia that makes vascular walls vulnerable to trauma and rupture, causing skin and mucosa bleeding. It is of dominant autosomal inheritance, characterized by recurrent epistaxis and telangiectasia on the face, hands and oral cavity; visceral arteriovenous malformations and positive family history. Epistaxis is often the first and foremost manifestation. It's associated to arteriovenous malformations in several organs. There are possible hematologic, neurologic, pulmonary, dermatologic and gastrointestinal complications. Treatment is supportive and helps prevent complications. This study is a case report of a patient with this syndrome who came to the ENT Outpatient Ward of the Faculdade de Medicina de Marília; and we have done a bibliographic review of the disease's etiopathogenesis, clinical manifestations and clinical-surgical treatment options.

## INTRODUCTION

The Rendu-Osler-Weber syndrome or Hereditary Hemorrhagic Telangiectasia is a rare systemic fibrovascular dysplasia which bears, as basic defect, an alteration in the elastic and muscle layers of vessel walls, making them more vulnerable to spontaneous ruptures and injuries^1^, ^2^.

The disease is autosomal dominant, although in about 20% of the cases, there is no family history. The incidence in the general population is of 1-2/100,000 and has a homogeneous race and gender distribution^3^.

Diagnosis is made according to the Curaçao Criteria: telangiectasia on the face, hands and oral cavity; recurrent epistaxis; arteriovenous malformations with visceral involvement; family history. Diagnosis is confirmed upon the presence of at least 3 of these manifestations^4^.

Otorhinolaryngological manifestations are the most frequent, and recurrent epistaxis is the main complaint. Blood vessels from other regions may also be involved, especially those from the lungs, brain, skin and gastro-intestinal tract^1^, ^4^, ^5^, ^6^.

Treatment is of palliative nature only; there still is no consensus on the best treatment option. The important thing here is to control the disease for as long as possible. Options vary between anterior and posterior nasal packing and chemical or laser cauterization of the lesions, all the way to surgical treatment, such as dermoseptoplasty and nasal cavity obliteration through Young's approach^5^, ^6^. With the progress in diagnostic image tests, today it is possible to embolize the Maxillary, Ethmoidal or Sphenoidal arteries, as well as ligation of the Maxillary or Palatine arteries in recurrent cases, in an attempt to contain bleeding and improve the patient's anemia^5^.

Because of this lack of consensus regarding the best treatment for these cases, all cases should be described so as to provide a better understanding of this disease. This paper also aims at alerting otolaryngologists that, when facing a patient with recurrent epistaxis, one must suspect of Rendu-Osler-Weber syndrome, and perform a nasal fibroscopy to better diagnose this syndrome.

The present investigation reports the case of one patient with Rendu-Osler-Weber syndrome; seen at the ENT Ward at Faculdade de Medicina de Marília and we review the literature and discuss available treatment options.

## CASE PRESENTATION

C.L.S., 53 years old, came to the Medical School of Marília Department of Otorhinolaryngology, complaining of mild recurrent epistaxis for 15 years, with worsening of the clinical Picture in the past 3 months, having had bleeding for more than one hour three times a day, always after physical exercise or sneezes. So far the patient had not had the need for nasal packing or other treatment.

**Figure 1:**
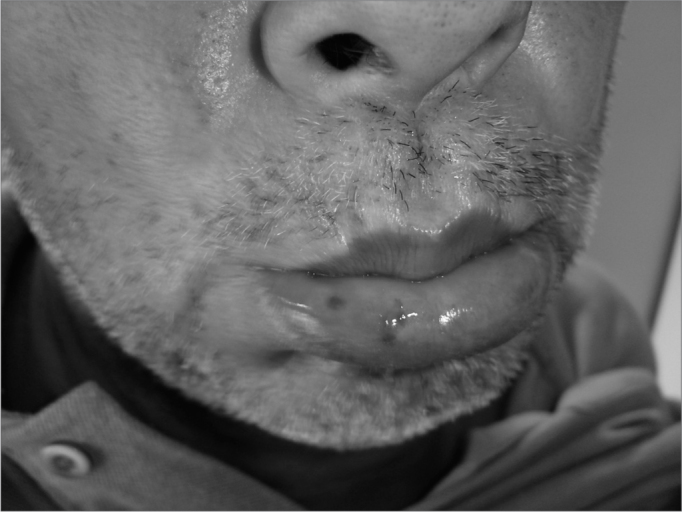
Lower lip Telangiectasia.

**Figure 2:**
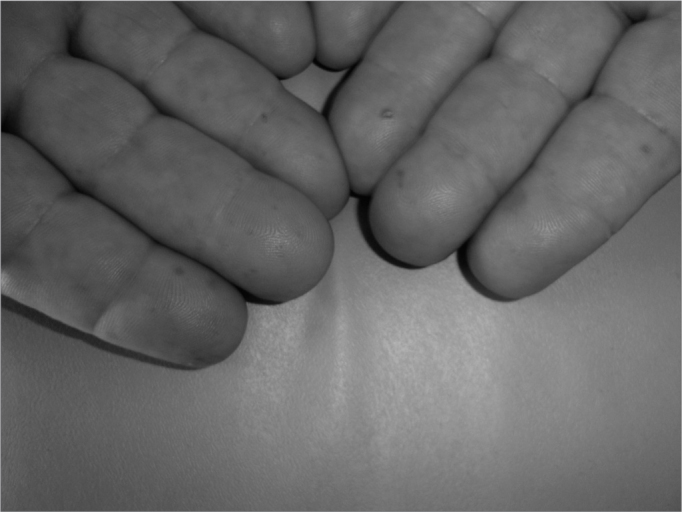
Fingers Telangiectasia.

For about 30 years he had hemangiomas spread throughout his body, especially in the chest, lower lip (Figure 1), tongue, cheek mucosa, face, fingers and nail bed. (Figure 2). He was also hypertensive and used captopril 25mg bid and propanolol 40mg bid. He did not use salicylate-based drugs. His father had repetition epistaxis, however milder and with telangiectasia lesions on his skin and mucosa, without visceral lesions; and he had an 8 year old grandson with mild epistaxis.

During his physical exam, we noticed telangiectasia lesions spread throughout his chest, nails, fingers, lower lip, oropharynx, tongue and face. Doing anterior rhinoscopy, we noticed hematic points on his septal mucosa and his left middle turbinate. There were also hematic crusts on his right septal mucosa.

His chest x-ray, abdominal echography and skull and abdomen CT scan did not show arteriovenous malformations. Blood tests showed Hb 8.3g/dl, Ht 26.6%.

As first treatment, we packed his nose and admitted him to the hospital for blood transfusion. After we removed the nasal packing, the epistaxis episodes relapsed, and we then decided to cauterize the nasal lesions through nasal endoscopy. Finally, we carried out right side maxillary artery embolization with an improvement in the intensity of the epistaxis episodes, however without complete remission. The patient remains under follow up in an outpatient basis and has been referred to Hematology, Gastroenterology and Thoracic Surgery for evaluation and they found no pulmonary and gastrointestinal malformations. He is still being followed up in the hematology ward treating anemia and using aminocaproic acid since then. His last blood test showed Hb 12.4g/dL and Ht 37.4%.

## BIBLIOGRAPHIC REVIEW AND DISCUSSION

### Background

This disorder was first described in 1864, by Henry Gawen Sutton (1836 - 91), who reported on a disorder that caused epistaxis, skin telangiectasia and internal bleeding^7^. One year afterwards, Benjamin Guy Babington (1794 - 1866) described repetition epistaxis in 5 generations of a family, and he was a Pioneer in describing the hereditary characteristic of this disease^4^. In 1876, John Wickham Legg (1843 - 1921) also described this disease, but just like Chiari in 1887 and Chaufffadin, in 1896, he was not able to differentiate this disease from hemophilia^2^, ^4^.

In was only in 1896, that Louis Marie Rendu (1844 - 1902) published a description of a 52-year-old man with recurrent epistaxis and telangiectasia on his face and torso, and lesions on his lips and soft palate. He was the first to suspect of nasal lesions causing epistaxis. He also noticed the presence of nasal bleeding in the patient's mother and brother^4^.

In 1901, Wiliam Osler (1849 - 1919) reported three cases in which he described the disease's hereditary characteristic; he was also the first to report that viscera could be affected^4^, ^5^, ^6^.

In 1907, Frederick Parkes Weber (1863 - 1962) reported a series of patients in whom he noticed lesions on their fingers, especially under the nails. It was in 1909 that Hanes coined the term Hereditary Hemorrhagic Telangiectasia, however, the disease is known today by the eponym of Rendu-Osler-Weber's syndrome^4^.

### Genetic Heritage

It is a hereditary disorder, with dominant autosomal transmission, despite the fact that about 20% of the cases do not have a family history - they could represent sporadic mutations^7^.

It occurs in heterozygotes with incomplete expression in some cases. The homozygote form is not compatible with life^6^. Many genes have been identified and implicated with the disease's pathogenesis; however, the most important ones so far are the Endoglin (ENG) in chromosome 9 and the type II A Activin - Como I (ACVRL-1) receptor, being the former associated with a variant with pulmonary manifestations, known as THH1 and the latter with a more subtle phenotype of late onset^8^. Both genes code a membrane glycoprotein that is specially expressed in endothelial tissue cells and make up the surface receptor for the β growth factor (TGF-β), which will mediate vascular remodeling by affecting the extracellular matrix production^8^, ^9^. ENG, ACVRL - 1 and TGF-β function are essential for angiogenesis^8^, ^9^.

### Histopathology

The histological findings once described by Jahnke in 1970 were an increase of submucosal vessels in intact endothelium, dilations of capillary, post-capillary and collector-type venules, with large elongated clusters of erythrocytes with fibrin channels in the connective tissue, red cells spread in the interstice around affected vessels, endothelial discontinuity and degeneration^2^, ^5^, ^10^. The first to describe these alterations was Menefee et al., in 1975, after a study with electron microscopy^10^.

The endothelial cells described were of three types: Normal - 90% of them; Degenerative - 7%, with a dense cytoplasm and less organelles present. They are less adherent to adjacent cells and develop slots between them; Cuboids - 3% found at random^10^. The basal lamina of these cells were intact, sometimes doubled, even where the cells were degenerated or there were slots between them. Associated with a reduplication of the basal lamina, we observed frequent microfibrillae in the connective tissue, without other clear alterations^2^.

In 1990, Braverman, studied the development of skin telangiectasia. In a normal skin, the arterioles are connected to the venules through multiple capillaries in the papillary dermis region. These vessels come from other larger vessels at the skin-fat tissue junction. The post-capillary venule's normal ultra-structure includes the lumen (L), endothelial cells and two or three layers of adventitious cells. At first, the simple venules become dilated; however they are still connected to the arterioles by means of one or more capillaries. There is a perivascular lymphocytic infiltrate. In a more advanced stage, the venules and their branches are markedly dilated, elongated and rolled in the dermis. The connection arterioles are also dilated and such dilation makes the capillaries disappear after some time, causing the arteriovenous malformations (shunts and fistulas). The perivascular lymphocytic infiltrate is still present. The vessel walls loose their elastic fibers, while the endothelial layer, the basal membrane and the smooth muscles remain intact^11^.

### Diagnosis

Clinical diagnosis is made based on the Curaçao Criteria, established by the Scientific Division of the Hereditary Hemorrhagic Telangiectasia International Foundation. It is based on:

1- epistaxis - spontaneous and recurrent nasal bleeding;
2- telangiectasia - multiple and in characteristic sites (lips, oropharynx, fingers and nose)
3- visceral lesions - such as gastrointestinal telangiectasia with or without bleeding; pulmonary, hepatic, brain and spinal arteriovenous malformations;
4- family history - one first degree relative with Hereditary Hemorrhagic Telangiectasia^4^.

The multiple clinical manifestations associated with Hereditary Hemorrhagic Telangiectasia involve nasal, skin, pulmonary, cerebral and gastrointestinal tract vascular abnormalities^5^, ^6^.

### Clinical Manifestations

 

### Nasal

Epistaxis caused by spontaneous bleeding of nasal mucosa telangiectasia is the most common manifestation of this disease; however, it does not happen to all patients, about 80% of them have recurrent epistaxis^5^. Disease severity varies from severe epistaxis that requires multiple blood transfusions and oral iron supplementation to such mild manifestations that the disease is never suspected^6^. Bleeding starts around 10 year of age in some patients and before 21 years of age in almost all; however, they become more severe in later decades in two-thirds of the patients^2^.

### Skin

The most characteristic lesion is the macular telangiectasia, with about 2 millimeters in diameter, which affect the face, lips, nose, tongue, ears, hands, upper body and feet^2^, ^5^. They usually manifest later than epistaxis, and are detected around the third decade of life^6^. These lesions may bleed, however the bleeding is not clinically relevant.

### Pulmonary

These are arteriovenous malformations causing direct communication between the pulmonary vein and artery by means of a thin-walled aneurism^2^. They are multiple and appear in both lungs, having a predilection for the lower pulmonary lobes. It is estimated that about 60% of the persons with pulmonary arteriovenous malformations have Hereditary Hemorrhagic Telangiectasia; however, between 15 and 33% of the persons with such disease have pulmonary arteriovenous malformations, although such incidence vary according to the specific gene involved^9^.

They produce a right-to-left shunt^6^. Symptoms start around the third or fourth decade of life and patients may, depending on shunt severity, present with profound dyspnea, fatigue, cyanosis or polycytemia^6^. Nonetheless, initial manifestations usually are sequela of neurologic lesions such as ischemic stroke and cerebral abscess.

They may be found as a holosystolic extracardiac murmur during deep inspiration or by means of image exams^6^. Chest X-Ray shows a typical mass with artery and vein enlargement, however, the lesions may be found in the posteroinferior lung region^2^, ^5^. Chest CT-scan must be used and pulmonary angiography should be used only for radiological or surgical treatment planning^6^.

### Cerebral

Results from pulmonary arteriovenous fistulas in about 60% of the cases, cerebral malformations in about 28%, spinal chord malformation in 8% and portasystemic encephalopathy in 3%^5^, ^6^.

They happen in between 8 and 12% of the patients and symptoms include: headaches, vertigo, syncope, visual and hearing disorders, focal and generalized crisis, obnubilation and paraparesias^6^.

Brain abscesses, Ischemic Strokes, Bacterial encephalitis happen only in patients with right-to-left shunt and pulmonary arteriovenous malformations, which facilitate the passage of septic emboli to the brain circulation^2^.

### Gastrointestinal Tract

In about 10% of the cases there is recurrent and small high and low gastrointestinal tract bleeding1. They happen mainly between the fifth or sixth decades of life and are caused by mucosal telangiectasia similar to those that happen in the oral and nasal mucosa^1^, ^4^, ^5^. In half of the patients, the bleeding cause may be located in the stomach or duodenum^5^. There may be larger telangiectasia, angiodysplasia or less commonly, arteriovenous malformations^2^. There are reports of a greater incidence of duodenal ulcer; however, without clinical correlation with the disease^12^.

Liver involvement is rare. An arteriovenous malformation between the hepatic vein and artery may produce a left-to-right shunt, which may cause congestive heart failure^2^, ^5^. A shunt between the hepatic and porta vein may cause hepatic encephalopathy and gastrointestinal bleeding. Malformations between the hepatic artery and the porta vein may cause portal hypertension with esophageal varicose veins. This may be suspected when there is hepatomegalia or a noise over the liver. Other findings include a mild increase in alkaline phosphatase, γ-GT, and cholestasis. Ultrasound and CT scan may be used for diagnostic purposes ^6^.

Another type of liver involvement is hereditary hemorrhagic telangiectasia cirrhosis, characterized by dilated vessels surrounded by a variable amount of stroma and randomly distributed throughout the liver. Within these structures we have normal liver tissue^6^.

### Treatment

Treatment is rarely necessary for skin lesions. However, when the telangiectasia cause cosmetic changes or bleed frequently, laser ablation or topic agents may be used^5^.

In pulmonary malformation, the classical treatment is surgery, however it is invasive and must remove as little as possible from the healthy pulmonary tissue around the malformation. The current treatment of choice is embolization of the vessels found, although there are still no studies regarding the long term follow up of these patients^9^.

The treatment of small gastrointestinal bleedings may be carried out by a small combined dose of estrogen and progesterone that, according to Carney^13^, has reduced the need for transfusion even for some months after the end of treatment. Results with endoscopic laser and bipolar coagulation are disheartening, because the lesions, especially those in the small bowel, are not easily found^13^. Aminocaproic acid has been suggested as a treatment option; however its efficacy has been questioned^13^.

Treatment of hepatic malformations is conservative^14^. Experience with surgical ligation is limited and data on embolization, although limited, show mortality in about 25% of the cases^14^.

Regarding malformations in the nervous system, there is no consensus as to the best treatment option - conservative or surgical, thus the decision depend on surgical risks and lethal bleeding, as well as surgeon's experience and lesion location. Different treatment modes such as embolization and estereotactic radiosurgery with γ rays may be used alone or in combination^6^.

And finally, this study highlights epistaxis control. There is no standard treatment^1^, ^15^. The multiple modes vary: anterior and posterior nasal packing, electric and/or chemical cauterization, vessel ligation, dermoseptoplasty, estrogen, hormonal therapy and, more recently, laser treatment^16^. However, no treatment has obtained full success and often times it is necessary to use more than one type of treatment to control the epistaxis^1^, ^2^, ^16^.

Treatment aims at reducing the number of bleeding episodes, as well as their intensity^16^. It tries to reduce the need for blood transfusions and hospitalizations^1^, ^2^. It also aims at improving the quality of life for those patients, who already suffer from constant bleeding episodes^1^, ^2^.

In general, treatment with cauterization, local pressure or packing is only palliative, and must be used in acute spells only^1^, ^2^. Arterial embolization, estrogen therapy or laser, Young's procedure and epithelial slice cultures are used as definitive treatment options in mild cases, bearing in mind that there is important bleeding recurrence and often times many treatment modalities are carried out^16^. Dermoseptoplasty and local microvascularized flaps are saved for the most severe cases^16^.

Hormonal treatment is carried out with high doses of estrogen, inducing nasal mucosa metaplasia towards a thick layer of keratinized squamous epithelium, which cover eventual nasal lesions, thus protecting them from local trauma^2^. Although of proven efficacy, hormonal therapy has not been broadly used because of its potential side effects - nausea, mammary sensitivity, weight gain and coronary diseases^6^. Moreover, male patients may develop gynecomastia, testicular atrophy and loss of libido^1^, ^2^.

Surgical treatment for this disease include: dermoseptoplasty and nostril closure by Young's technique^16^. Dermoseptoplasty or Saunders's procedure was described by the former in 1962, and used a thin skin layer to cover the anterior area of the nose. Skin grafts may be harvested from the thigh or arm, labial mucosa or amniotic membrane. Surgery consists of removing the affected mucosa and placing the flat over it, covering the nasal septum and inferior turbinates, bilaterally^17^. It is considered gold standard in the treatment^1^, ^2^, ^6^, ^13^. Nostril closure according to Young's procedure is carried out through an incision in the mucocutaneous junction of the nasal vestibule, which is taken backwards. It brings about severe side effects, such as oral breathing, and it is considered a last resort in very well selected cases^17^.

Microembolization is carried out using gelfoam or sclerosing substances from the maxillary artery under visualization of angiographic films^16^. Brachytherapy and fibrin glue injection in the nasal septum and inferior turbinates submucosa are efficacious, however they bring about only temporary symptom improvement^18^. Treatment with aminocaproic acid is not very much used; however it brings about significant symptoms improvement. Its side effects are: nausea, cramps, diarrhea, hypotension, dizziness, skin rash, myopathy, fatigue, rabdomyolysis, renal dysfunction and thrombosis^16^.

Laser is saved for acute bleeding spells, in an attempt to contain those. In the literature we have reports on CO_2_, o Nd-YAG, Argon, KTP and pulsed-dye lasers. It is difficult to compare these lasers because of a non-homogeneous classification in the current world medical literature^1^, ^2^, ^16^.

## FINAL COMMENTS

Hereditary Hemorrhagic Telangiectasia is a multisystemic disease, first manifested by repetition epistaxis. Thus, it is fundamentally important that the otorhinolaryngologist be up-to-date in relation to the disease's etiopathogenesis and treatment options, so as to perform correct diagnosis as well as to prevent systemic complications of this disease.

Thus, this paper aimed at providing an update on the disease, as well as report a clinical case from the ENT Ward of the Medical School of Marília - Faculdade de Medicina de Marília, since there are treatment options, but we still lack a totally satisfactory treatment modality. It is very important to record all cases found in order to compare clinical manifestations and treatment approaches so that in the near future we may create Standards with effective results and life quality improvement for the patients.
